# Vaccinomics Approach for Multi-Epitope Vaccine Design against Group A Rotavirus Using VP4 and VP7 Proteins

**DOI:** 10.3390/vaccines11040726

**Published:** 2023-03-24

**Authors:** Muhammad Usman, Aaima Ayub, Sabahat Habib, Muhammad Suleman Rana, Zaira Rehman, Ali Zohaib, Syed Babar Jamal, Arun Kumar Jaiswal, Bruno Silva Andrade, Vasco de Carvalho Azevedo, Muhammad Faheem, Aneela Javed

**Affiliations:** 1 Atta-ur-Rahman School of Applied Biosciences, National University of Sciences and Technology, Islamabad 44000, Pakistan; 2 Department of Virology, National Institute of Health, Islamabad 45500, Pakistan; 3 Department of Microbiology, The Islamia University of Bahawalpur, Baghdad-ul-Jadeed Campus, Bahawalpur 63100, Pakistan; 4 Department of Biological Sciences, National University of Medical Sciences, Rawalpindi 46000, Pakistanmuhammad.faheem@nums.edu.pk (M.F.); 5 Laboratory of Cellular and Molecular Genetics (LGCM), PG Program in Bioinformatics, Department of Genetics, Ecology, and Evolution, Institute of Biological Sciences, Federal University of Minas Gerais (UFMG), Belo Horizonte 31270-901, Brazil; 6 Laboratory of Bioinformatics and Computational Chemistry, State University of Southwest of Bahia, Bahia 45083-900, Brazil

**Keywords:** acute gastroenteritis, viruses, vaccinology, rotavirus, multivalent, in silico

## Abstract

Rotavirus A is the most common cause of Acute Gastroenteritis globally among children <5 years of age. Due to a segmented genome, there is a high frequency of genetic reassortment and interspecies transmission which has resulted in the emergence of novel genotypes. There are concerns that monovalent (Rotarix: GlaxoSmithKline Biologicals, Rixensart, Belgium) and pentavalent (RotaTeq: MERCK & Co., Inc., Kenilworth, NJ, USA) vaccines may be less effective against non-vaccine strains, which clearly shows the demand for the design of a vaccine that is equally effective against all circulating genotypes. In the present study, a multivalent vaccine was designed from VP4 and VP7 proteins of RVA. Epitopes were screened for antigenicity, allergenicity, homology with humans and anti-inflammatory properties. The vaccine contains four B-cell, three CTL and three HTL epitopes joined via linkers and an N-terminal RGD motif adjuvant. The 3D structure was predicted and refined preceding its docking with integrin. Immune simulation displayed promising results both in Asia and worldwide. In the MD simulation, the RMSD value varied from 0.2 to 1.6 nm while the minimum integrin amino acid fluctuation (0.05–0.1 nm) was observed with its respective ligand. Codon optimization was performed with an adenovirus vector in a mammalian expression system. The population coverage analysis showed 99.0% and 98.47% in South Asia and worldwide, respectively. These computational findings show potential against all RVA genotypes; however, in-vitro/in-vivo screening is essential to devise a meticulous conclusion.

## 1. Introduction

Diarrhea is reported to be one of the primary reasons for death in children of age less than five years. It is responsible for causing half a million deaths every year around the globe. The estimated number of deaths due to Rotavirus in the year 2000 was around 528,000 which has declined to 185,300 deaths annually. A total of 85% of these deaths were recorded in low-income countries. This decline in deaths may be due to revised estimates of overall deaths as well as due to the introduction of the Rotavirus vaccine [1]. 

Rotaviruses (RV) belong to the family of Reoviridae and have been detected in different organisms including birds and mammals [2]. The non-enveloped virus has three layers of capsids and a double-stranded RNA genome of 11 segments [3,4,5]. Its genome encodes six structural proteins (VP1-VP4 and VP6-VP7) and six nonstructural proteins (NSP1-NSP6). Based on the VP6 protein Rotaviruses are classified into ten groups (A-J) also known as species. Group A Rotaviruses most commonly infect humans especially children <5 years of age and therefore are the primary choice for vaccine development [2,3].

Out of all these proteins, the two outer capsid proteins VP7 and VP4 bear special attention due to their important role in the rotavirus genome characteristics. Both these proteins are serotype cross-reactive and serotype-specific, which can elicit a neutralizing antibody response [6,7]. Likewise, the current Rotavirus classification system is also based on these two proteins: VP7 is a glycoprotein which is also named as G-Type while VP4 is a protease-sensitive protein, also named P-Type. Until now 41 G-Types and 57 P-Types have been identified globally. Moreover, these two proteins have been selected as a target for vaccine preparation for many years due to their considerably large-sized surface-exposed structure [8,9].

To counter the colossal rate of RV infections, a live attenuated vaccine named Rotashield was introduced in 1998 but soon due to intussusception cases and the poor effectiveness of the vaccine, it was taken off. However, this vaccine gave way to many more attempts [10,11]. In 2009, the WHO recommended introducing the two live attenuated RVA Vaccines (RotaTeq: Merck & Co, Rotarix: GlaxoSmithKline) into their routine immunization program globally [12]. Currently, 123 countries have already introduced the vaccine into their population including Pakistan which has introduced the Rotarix vaccine into its Extended Program on Immunization (EPI) initially as a phased introduction at a subnational level in five districts of the province of Punjab and then at the national level in 2018 [13,14]. RotaTeq is a pentavalent vaccine (G1P [5], G2P [5], G3P [5], G4P [5], and G6P [8]) while Rotarix is a monovalent vaccine (G1P [8]). Vaccine efficacy of RotaTeq is reported to be 45% in higher mortality rate countries and 90% in lower mortality rate countries, while the Rotarix vaccine efficacy is reported to be 57% in high mortality rate countries, 75% in medium and 84% in lower mortality rate countries [15].

The segmented genome nature of RVA and its multiple host range causes a high frequency of genetic reassortment and interspecies transmission to form a novel genotype [16]. Similarly, vaccine-induced immune pressure can also be involved in the emergence of novel RV strains which may cause serious illness even in vaccinated children [17]. Therefore, the efficacy of live attenuated vaccines to provide immunity against all G and P types as well as against any unusual strain in case of an outbreak is of great question [18]. This genetic variation of Rotaviruses is a consequence of repeated infection in children, especially in impoverished settings. A study conducted in India followed the children from birth for three years and concluded that 63% of children who suffered from RV experienced at least three infections before reaching their third birthday [19]. To deal with this problem, a highly conserved sequence-specific vaccine target should be designed to gain effective vaccine strategies.

Although live attenuated vaccines (Rotarix, RotaTeq) produce a strong immune response and do not require multiple doses, there is a probability of reversion to a more pathogenic strain, a lower efficacy against non-vaccine strains, limitations to administering the vaccine to immunocompromised individuals and strict storage conditions making the vaccine compromised. Likewise, it also has been reported that the risk of intussusception is 5.7-fold increased after the first dose of RV vaccine [20]. On the other hand, the peptide vaccine has minimum or no side effects, can also produce a strong immune response when designed with an adjuvant, is safe to administer to immunocompromised individuals and does not require low temperature storage conditions [21,22].

To counter the downsides of traditional vaccines, immunoinformatic approaches have been widely utilized for the in silico development of vaccines against a wide range of organisms such as Ebola virus, Avian Influenza virus, Dengue virus, Trypanosoma cruzi and many others [23]. The current multiepitope based vaccine was designed to find out effective B-cell and T-cell epitopes against RVA by using available VP4 and VP7 genotype sequences prevalent globally. During the in silico analysis, the vaccine construct exhibited an innate and humoral response against heterotypic genotypes, but in vitro and in vivo analyses are needed to further confirm our in silico results.

## 2. Materials and Methods

### 2.1. Rotavirus Sequence Retrieval and Multiple Alignments

**Figure 1:**
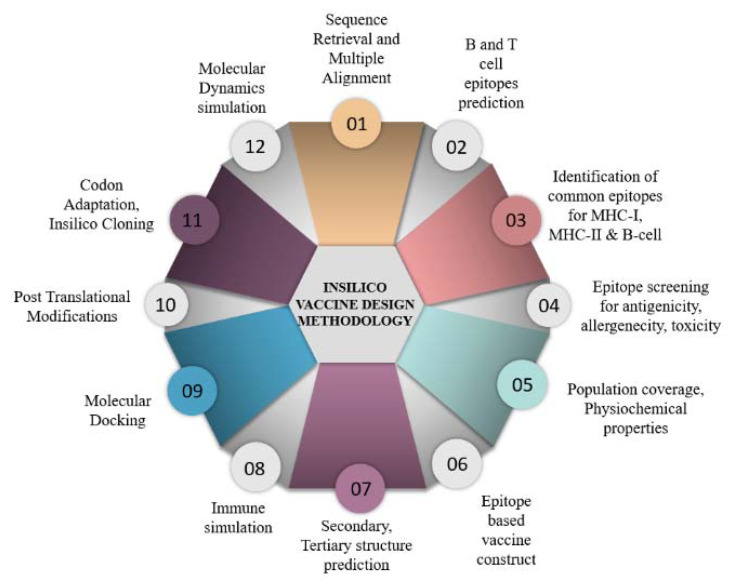
Schematic representation of the strategy followed for the in silico vaccine design against RV.

A schematic representation of the detailed methodology is presented in Figure 1. The amino acid sequences of structural proteins (VP4 and VP7) of Rotavirus group A were downloaded from the NCBI (https://www.ncbi.nlm.nih.gov/genbank/, accessed on 2 March 2023). All the globally reported sequences of VP7 and VP4 genotypes were included in the study.

Multiple sequence alignments were performed separately for individual serotypes of VP4 and VP7. For each serotype, the consensus sequence was generated. All the consensus sequences were then subjected to alignment to generate the final consensus sequence of VP4 and VP7. MAAFT (https://www.ebi.ac.uk/Tools/msa/mafft/, accessed on 2 March 2023) [24] was used to perform multiple sequence alignment and the consensus sequence was generated through CLC Genomics Workbench 8.5.1 (CLC Bio, Aarhus, Denmark). The consensus sequences of VP4 and VP7 were further subjected for epitope identification.

### 2.2. T-cell Epitope Prediction 

In identifying the vaccine epitopes, T-cell epitope prediction is detrimental. Among T-cell epitopes, cytotoxic and helper T-cells epitopes play an important role in eliciting immune responses. Hence, T-cell epitopes with the binding affinity for MHCI and MHCII were identified. The CTL epitopes had a binding affinity with MHC1 which was predicted through NetMHCpan4.1 (https://services.healthtech.dtu.dk/service.php?NetMHCpan-4.0, accessed on 2 March 2023) while T-helper cell epitopes had a binding affinity with MHC-II which was predicted via NetMHCpan2 (https://services.healthtech.dtu.dk/service.php?NetMHCIIpan-4.0, accessed on 2 March 2023) [25]. NetMHCpan4.1 predicted the binding of different human MHC alleles HLA-A, -B, -C, -E by employing the artificial neural network. On the other hand, NetMHCpan2 predicted the affinity for HLA-DR, -DQ, and –DP. Most of the HLA molecules tended to bind with 9-mer epitopes. Owing to this property of HLA molecules, 9-mer epitopes were chosen as the target length of predicted epitopes. The resulting epitopes were screened for 70% conservancy to achieve epitopes from sites with fewer variations using Unipro UGENE (http://ugene.net/, accessed on 2 March 2023) [26].

### 2.3. B-Cell Epitope Prediction and Common Epitope Identification

The linear B-cell epitopes were predicted through ABCPred (https://webs.iiitd.edu.in/raghava/abcpred/ABC_submission.html, accessed on 2 March 2023) [27]. The ABCPred prediction method is based on recurrent neural network (RNN) and the standard feed-forward (FNN). The 20 amino acids long peptides with default threshold of 0.51 were processed further. The overlapping B-cell epitopes with MHC -I and MHC-II epitopes were shortlisted for screening. 

### 2.4. Feature Profiling of Epitopes 

The primary parameters for epitopes to be acceptable as a potentially viable vaccine construct are their ability to be antigenic enough to evoke an immune response, be non-allergen, non-toxic, anti-inflammatory and non-homologous to human proteins. A higher population coverage of T cells can be indicative of a good vaccine. The antigenicity of the final selected epitopes was determined using the VaxiJen (http://www.ddg-pharmfac.net/vaxijen/VaxiJen/VaxiJen.html, accessed on 2 March 2023) [28], allergenicity was determined by AllerTop (https://www.ddg-pharmfac.net/AllerTOP/, accessed on 2 March 2023), the toxicity through Toxinpred (http://crdd.osdd.net/raghava/toxinpred/, accessed on 2 March 2023) [29,30] and the anti-inflammatory response was predicted by AIPpred (http://www.thegleelab.org/AIPpred/, accessed on 2 March 2023). The nonhuman homologs were predicted through blast-p (https://blast.ncbi.nlm.nih.gov/Blast.cgi?PAGE=Proteins, accessed on 2 March 2023) [9]. The epitopes with <30% identity with human proteome were selected. The population coverage of T-cells worldwide, in Southeast Asia and in Africa was calculated by the IEDB Population coverage tool (http://tools.iedb.org/population/, accessed on 2 March 2023) [31]. The epitopes fulfilling all the properties were further subjected to the development of the vaccine construct.

### 2.5. Development of Epitope-Based Vaccine Construct

**Figure 2:**
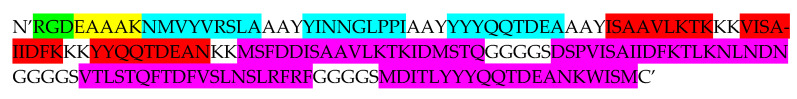
The sequence of the vaccine construct. (Green: Adjuvant, Yellow: Linker for attaching adjuvant to CTL; Cyan: Cytotoxic T-cell epitopes; Magenta: Helper T-cell epitopes; Red: B cell. Uncolored residues include AAY linker between CTL epitopes, KK linker between HTL epitopes, and GGGGS linker joining the B cell epitopes.

To develop a vaccine construct, the N-terminal integrin-binding motif (Arginine-Glycine-Aspartate (RGD)) was used as an adjuvant. The EAAAK linker was used to join the adjuvant with CTL epitopes, the AAY linker was used between CTL epitopes, the KK linker was used between HTL epitopes, and the GGGGS linker joined the B cell epitopes [32]. The final construct is shown in Figure 2.

### 2.6. Secondary and Tertiary Structure Prediction and Validation of the Designed Construct

The secondary structure of the final vaccine construct was predicted via PSIPRED and RaptorX Property servers [33]. The three-dimensional structure of the vaccine construct was predicted through trRosetta (https://yanglab.nankai.edu.cn/trRosetta/, accessed on 2 March 2023) [34] and validated via the ERRAT server (https://saves.mbi.ucla.edu/, accessed on 2 March 2023) [35] Procheck (https://saves.mbi.ucla.edu/, accessed on 2 March 2023) 3D refine (http://sysbio.rnet.missouri.edu/3Drefine/, accessed on 2 March 2023) and MolProbity tool (http://molprobity.biochem.duke.edu/, accessed on 2 March 2023) [36]. Physiochemical properties of individual epitopes of the whole vaccine construct with the adjuvant and without the adjuvant (not shown) were determined through ProtParam (https://web.expasy.org/protparam/, accessed on 2 March 2023).

### 2.7. Immune Simulation

Immune simulation analysis gave a visual of the response evoked by the vaccine in the body. Interactions of innate and adaptive immune system components were analyzed in a position-specific scoring matrix in C-IMMSIM (http://150.146.2.1/C-IMMSIM/index.php?page=1, accessed on 2 March 2023) tool [37]. Host HLA selection was completed based on scorings of alleles from epitope screenings and confirmed through the prior literature to select LA-A (HLA-A*02:01, HLA-A*30:01), HLA-B (HLAB*55:01, HLA-B*08:01), and HLA-II (DRB3_0101, DRB1_0101) H. The rest were default parameters.

### 2.8. Codon Optimization and In Silico Cloning

The vaccine construct was reverse-transcribed, and the codon was optimized for the human expression system through the Gensmart codon optimization tool (https://www.genscript.com/tools/gensmart-codon-optimization/confirm_success, accessed on 2 March 2023). The human embryonic kidney cell line (HEK293) was chosen as mammalian expression system. The SnapGene tool (https://www.snapgene.com/, accessed on 2 March 2023) was employed for in silico cloning using adenoviral plasmid (pShuttle-CMV) with PasI and BbsI restriction enzyme sites to be expressed in the mammalian cell line under the CMV promoter.

### 2.9. Post-Translational Modification (PTM)

The post-translational modifications (phosphorylation and glycosylation) of the vaccine polypeptide were investigated for the mammalian expression system. For N- and O-linked glycosylations, NetNGlyc (http://www.cbs.dtu.dk/services/NetNGlyc/, accessed on 2 March 2023) and NetOGlyc (http://www.cbs.dtu.dk/services/NetOGlyc/, accessed on 2 March 2023) servers were used while NetPhos (http://www.cbs.dtu.dk/services/NetPhos/, accessed on 2 March 2023) was used for the phosphorylation sites [38,39,40,41].

### 2.10. Molecular Docking

During an immune response, pattern recognition receptors (PRRs) play a vital role in stimulating an immune response [42,43]. Rotavirus permeates the cell membrane through the spike protein VP4 proteolytic cleavage which forms VP5 and VP8 fragments and together with the VP7 protein, these fragments interact with the α2 subunit of integrin to enter the cell [44]. 

Molecular docking was performed against integrin. The protein sequence of the α2 subunit of integrin (PDB ID: 2VDN) was retrieved from the RCSB database (https://www.rcsb.org/, accessed on 2 March 2023) and required monomers were selected using Chimera 1.14 (https://www.cgl.ucsf.edu/chimera/download.html, accessed on 2 March 2023) [45]. The docking was performed using ClusPro v2.0 (https://cluspro.bu.edu/login.php, accessed on 2 March 2023) [46] and visualized using BIOVIA, Dassault Systèmes, Discovery Studio, 2021 (https://www.3ds.com/products-services/biovia/products/molecular-modeling-simulation/biovia-discovery-studio/, accessed on 2 March 2023). 

### 2.11. Molecular Dynamics (MD) Simulation

We performed a molecular dynamics simulation for the integrin receptor using Gromacs 2018.8 package [47,48]. First, topology files were generated using the gmx module with the OPLS forcefield [49,50]. After that the complexes were solvated and neutralized with the TIP3P water model with a 1.0 nm distance. After the solvation and neutralization step, the energy minimization step was followed with one step of 50,000 cycles of energy minimization of steepest descent and conjugated gradient algorithms, in addition to one step of 1 nanosecond (ns) of volume equilibration and 1 ns of pressure equilibration. Then, the complex was submitted to an isobaric (1ATM) and isothermal (298,15 K) production for the molecular dynamics simulation run for 50 ns. The interaction and stability between each ligand-receptor complex were evaluated by generating graphs of ligand and receptor backbone RMSD, the RMSF of receptor amino acids, as well as the Coulombic and Lenard Jones interaction energy graphs.

## 3. Results

### 3.1. Rotavirus Sequence Retrieval and Selection of Epitopes for Multivalent Construct

**Figure 3:**
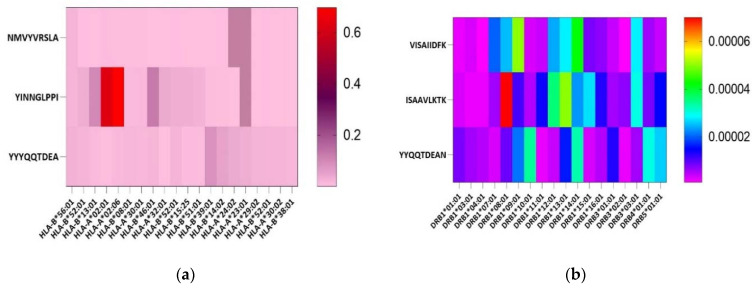
(**a**) Epitopes displaying a range of affinities to different human leukocyte antigen (HLA) Class I alleles. Red, purple and pink show strong, intermediate and weak, respectively. (**b**) Shows the affinity of screened epitopes with HLA Class II alleles. Pink shows the weakest while red indicates the highest binding score.

Based on the consensus sequence, 58,296 VP4 epitopes and 24,172 VP7 epitopes that bind with different MHC alleles in IEDB were retrieved. MHC-I peptides which were 70% conserved and common to all, MHC-II, IFN-gamma and B-cell, were shortlisted to give 11 epitopes for VP7 and 23 epitopes for VP4 which showed the highest interaction. After the rigorous screenings, a total of four B-cell, three HTL and three CTL epitopes fitted the desired criteria (Table 1) (Figure 3).

### 3.2. Epitopes Population Coverage across Asia, Worldwide and in Africa

**Figure 4:**
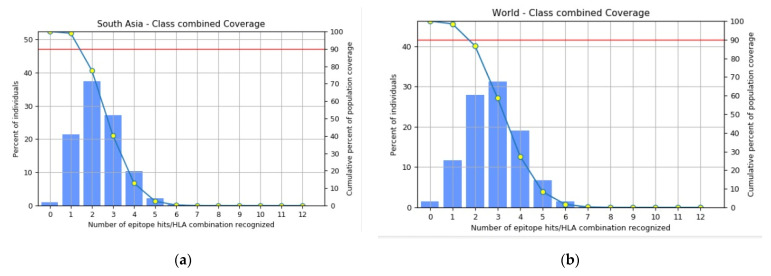
Percent class combined coverage of vaccine construct in (**a**) South Asia population and (**b**) worldwide population.

The population coverage analysis for selected B- and T-cell epitopes showed that these epitopes had 98.47% coverage worldwide and 99.0% coverage in the South Asian population (Figure 4, Table 2). The average hits for the worldwide and Asian populations were 2.82 and 2.33, respectively. Similarly, population coverage in different African countries ranged from 59.05% to 87.35% (Figure S3, Table 2).

### 3.3. Secondary Structure Prediction

**Figure 5:**
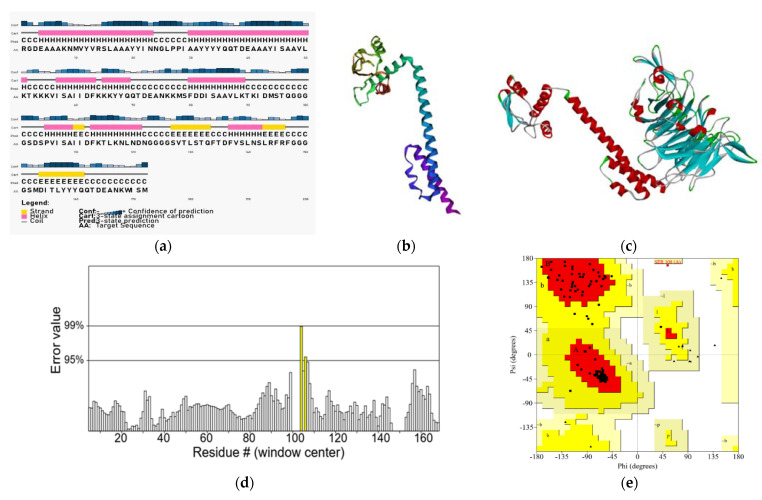
(**a**) The secondary structure of the vaccine. (**b**) 3D structure of the vaccine construct (from N (Purple) to C (Red) terminus) predicted by trRosetta) (**c**). Docked model of the vaccine construct (red) with an integrin receptor (cyan) visualized using BIOVIA, Dassault Systèmes, Discovery studio, 2021. (**d**) ERRAT scores of vaccine peptide. (**e**) Ramachandran plot of the vaccine peptide. A total of 93.5% residues in the favored region, 5.9% in the allowed region and only one residue in the disallowed region.

The final multi-epitope vaccine construct had 172 amino acids containing three CTL epitopes, three HTL epitopes and four B cell epitopes (Figure 2). The secondary structure analysis revealed that the vaccine peptide was composed of 52% α-helix, 8% β-strand and 38% coil. Solvent accessibility is a basic attribute that defines the stability of a structure. The exposed residues of a protein are more accessible to the solvent. The analysis of vaccine peptides showed that 25% of the amino acids were buried, 22% were medium exposed and 51% were exposed (Figure 5) which made them more accessible to the solvent. 

### 3.4. Physicochemical Properties Prediction

The molecular weight of the vaccine construct was 18878.30 g/mol, pI was 8.60, instability index (II) was computed to be 35.94, the grand average of hydropathicity (GRAVY) score was −0.291 and the protein was analyzed as stable. The estimated half-life was 1 hour in mammalian reticulocytes (in vitro), and 2 minutes in yeast and *Escherichia coli* (in vivo) (Table 3).

### 3.5. Molecular Docking with Integrin Subunit

*TrRosetta* generated five models of the vaccine epitope and the top scoring model with a TM score of 0.390 was selected for model validation (Figure 5d).

The refined model was docked with an integrin subunit (PDB ID: 2VDN). Model 0.00 was selected, and the lowest weighted score was −1172.4 Kcal/mol (Figure 5c) (Table 4).

### 3.6. Immune Stimulation for Vaccine Construct

**Figure 6:**
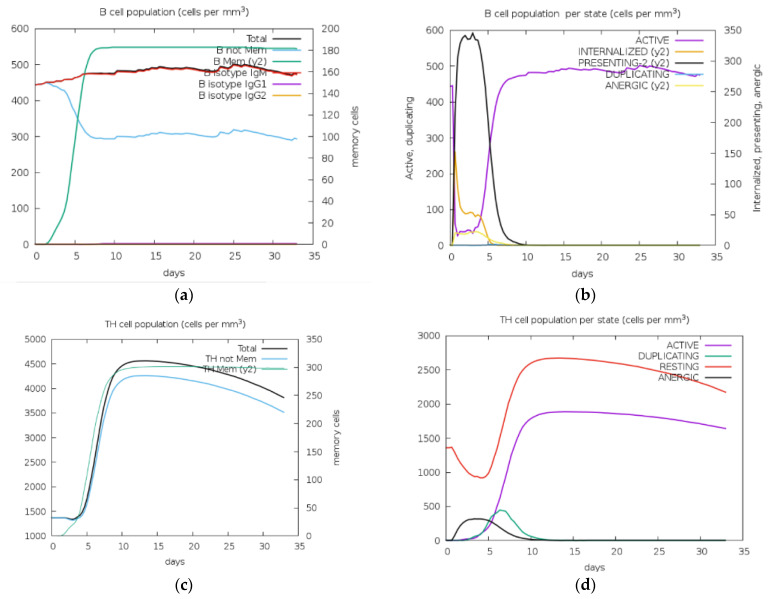

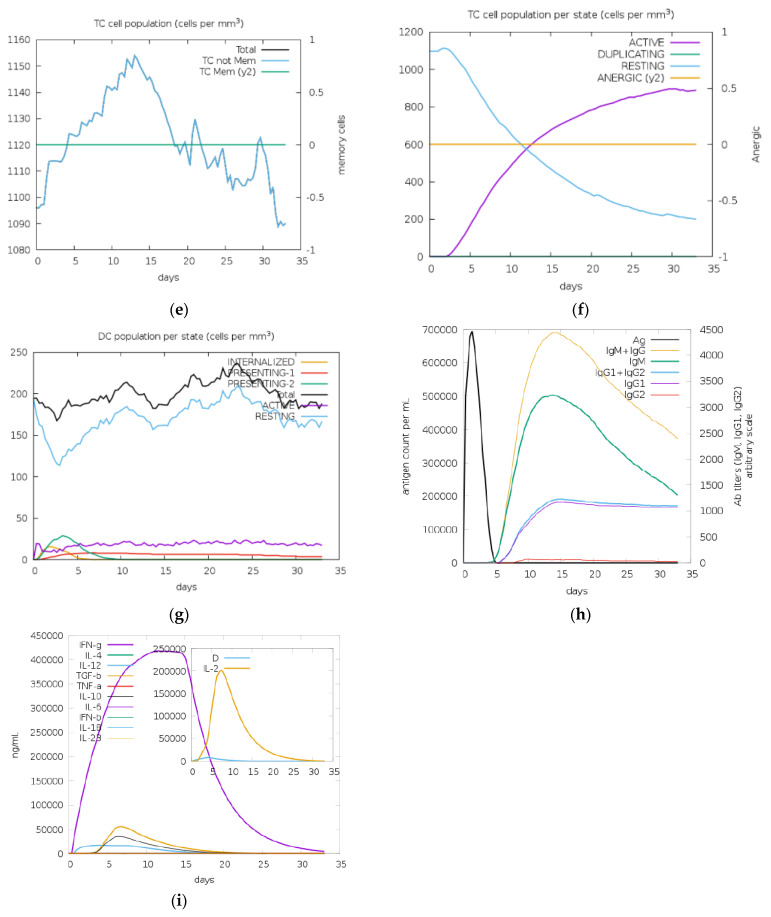
Immune simulation result of the vaccine construct showing clear immune system activity against the vaccine. Simulation of B cells (**a**,**b**), T cells (**c**–**f**), Dendritic cells (**g**), antibodies (**h**), and various cytokines (**i**). **IFN:** Interferon, **IgM/G:** Immunoglobulin M/G, **IL:** Interleukins, **TNF:** Tumor necrosis factor, **TGF:** Transforming growth factor, **TC cells:** Cytotoxic T cells, **TH cells:** T-helper cells. **Y2:** Scale of Memory Cells, **B Mem:** Memory B cells, **TH Mem:** Memory T-helper cells.

In silico immune simulation demonstrated the extent of the immune responses triggered by the designed vaccine construct. Desired immune responses were activated after injection including B-cell, T-cell (Cytotoxic), TH-cell (Helper), IgM and IgG production, and cytokines such as Interferon-gamma (Figure 6). The results successfully established that both adaptive and innate immune responses will be stimulated.

Codon optimization yielded the nucleic acid sequence of 516 base pairs with 54.26% GC content. These were cloned in silico using the SnapGene tool in the pShuttle-CMV vector (Figure S1).

### 3.7. Codon Optimization and In-Silico Cloning

Codon optimization through the Gensmart codon optimization tool in the human expression system yielded the nucleic acid sequence of 516 base pairs. The GC content of the multiepitope vaccine construct was found to be 54.26%. These were cloned in silico using the SnapGene tool in the pShuttle-CMV vector, an adenoviral plasmid vector, to be expressed in a mammalian cell line (Figure S1).

### 3.8. Post-Translational Modification (PTM) 

There were no potential N-glycosylation and O-glycosylation sites predicted in the vaccine construct whereas 39 phosphorylation sites were generated (15 serine, 11 threonine, 13 tyrosine) out of which 19 (8 serine, 8 threonine, 3 tyrosine) surpassed the threshold value, i.e., 0.5. 

### 3.9. Molecular Dynamics Simulation for Validation of the Tertiary Structure

The Ligand RMSD varied from 0.2 to 1.6 nm during all MD simulation times (Figure S2a). In this case, the molecule deviated from the integrin interaction since the first 0.5 ns of simulation time and increased above 1.4 nm until the end of the simulation. This indicated a huge variation which can be explained by the nature of the interactions between two proteins, ligand and receptor, indicating an accommodation of both molecules during the simulation time. 

Integrin amino acid fluctuations indicated lowest deviations below 0.1 nm, and reaching 0.05 nm, for the interaction region of the integrin receptor and its respective ligand from the residues ASP 245, GLIY 246, ASP 247, LEU 248, ASN249, ASP 301, ARG 335 and PRO337 (Figure S2b). 

Short-ranged Coulombic and Lenard Jones (LJ) interaction energies for the complexes maintained favorable values during all MD time, varying from −173.000 to −172.500 KJ/Mol for the coulombic interactions and −18.800 to −17.700 kJ/Mol for the as well (Figure S2c,d).

## 4. Discussion

The high burden of Rotavirus disease and mortality with a great diversity and distribution of RV strains differing from year to year and region to region show the clear need for an effective and safe vaccine which provides heterotypic protection against all circulating RV genotypes [51,52,53]. According to reports, the efficacy of Rotarix and RotaTeq vaccines against heterotypic RV genotypes is very low specially in low-income countries [54,55,56]. A shift in the RVA genotype has also been reported: G3P [8] and G2P [4] predominance after the introduction of RotaTeq and Rotarix vaccines, respectively, speculated as a result of immune pressure from the vaccinated population [53,54,55,56,57]. Immuno-informatics is one of the emerging fields in relation to vaccine design using reverse vaccinology especially in this era of evolving viruses and computational advances. Previous work on the in silico rotavirus vaccine has carried screenings of T-cell and B- cell epitopes which were used as a reference for this study [8,32,58,59,60]. 

The current multivalent vaccine was designed primarily against VP4 and VP7 proteins, against which neutralizing antibodies produced in the body were hypothesized to target all RVA genotypes circulating worldwide [61]. In previously reported studies, Devi et al. conducted a study on all structural and non-structural proteins of group A rotavirus, Dutta et al. narrowed it to VP7 and VP8 proteins, Jafarpour et al. limited theirs to VP4 and VP6 proteins, while, Shovu et al. were in accordance with our study in which they designed a vaccine against VP4 and VP7 proteins but of group A rotavirus circulating in Bangladesh only [8,32,58,59]. Moreover, the conservancy of their epitopes was up to 80% while the conservancy percentage of our epitopes was reduced to 70% since most of the 80% of conserved epitopes were lost in allergenicity and toxicity screenings. At 70% conservancy, epitopes were subjected to strict criteria of antigen screenings to shortlist 10 epitopes that were non-toxic, non-allergic, antigenic and induced B-cell, T-cell and interferon–gamma responses. 

Various HLA class I and class II alleles are associated with rotavirus susceptibility as discovered through studies of immune responses to vaccines [62,63,64,65]. Focally, the B-cell response is activated to counter rotavirus infection, demanding emphasis on its screening [66]. Four B-cell epitopes (MDITLYYYQQTDEANKWISM (VP7), MSFDDISAAVLKTKIDMSTQ (VP4), DSPVISAIIDFKTLKNLNDN (VP4), VTLSTQFTDFVSLNSLRFRF (VP4)), three CTL epitopes (YYYQQTDEA, NMVYVRSLA, YINNGLPPI) and three HTL epitopes (YYQQTDEAN, ISAAVLKTK, VISAIIDFK) were finalized for the multivalent vaccine construct. The population coverage on the construct revealed 98.47% coverage worldwide and 99% in South Asia, which was the main focus and showed a promising result. In comparison to these results, the vaccine designed by Shovu et al. only reached a population coverage of 45.64% for South Asia and 70.53% for the population worldwide, which highlights the efficacy of our vaccine [8].

Sana et al., and Chauhan et al. used B-defensin as an adjuvant, while Devi et al. chose a similar approach to our study by attaching N-terminal integrin-binding motif and Arginine-Glysine-Aspartate (RGD) as an adjuvant [32,67,68]. The synthetic peptide RGD binds to integrin, a receptor that grants susceptibility to cells for rotavirus, so its addition as an adjuvant improves the immunogenicity of the vaccine construct [69,70,71]. RGD motif and CTL epitopes were joined with an EAAAK rigid linker, an alpha helix forming linker which allows a fixed distance between them so they can maintain their independent qualities and boost immunogenicity [72].

Similar linkers were used in vaccine studies by Devi et al. for rotavirus, Chauhan et al. for SARS-CoV2, and Sana et al. for Crimean Congo hemorrhagic fever virus [32,67,68]. The principal function of linkers is to maintain the junctional immunogenicity between the epitopes. Cleavable linkers allow exposure of epitopes in the body upon cleavage, while flexible linkers permit movement. The instability index was calculated to be 35.94, which is less than threshold value 40, classifying the vaccine to be stable in vitro with the aliphatic index of 76.69 The findings in relation to the physicochemical properties demonstrate that our multivalent construct is a good candidate as a vaccine.

Unlike many studies that have used I-Tasser, Phyre and Raptor X for structure prediction, trRosetta was chosen for its rapid and accurate results for our multivalent construct [32,34,67,73,74,75]. Previous studies by Sana et al., and Devi et al. and various others utilized more than one tool for structure refinements such as GalaxyRefine, ModRefiner, and Yasara Energy Minimization Server; however, for this study, maximum ERRAT and Ramachandran scores were reached by use of 3Drefine only [32,67]. The final model was chosen based on the 3Drefine score, which shows the potential energy of the model, with the lowest value indicative of the best quality. The raw model was validated by the ERRAT score first before commencing refinement [67]. Before refinement, the model had a 98.701% ERRAT score, and Ramachandran favored residues of 92.94%. After refinement, the ERRAT score was 96.341%, and Ramachandran-favored residues were 96.47%. The values after refinement validate the vaccine to be of good quality. The ERRAT score and MolProbity parameters had definitive improvement before and after refinement with 3Drefine. Any structure with an ERRAT score greater than 80% is considered to be good, and since our vaccine construct reached an excellent value of 96.341%, no further refinement was necessary [8]. 

Immune simulation carried out in C-IMMSM revealed that the antigen titer fell drastically after 5 days, with a simultaneous increase in protecting antibodies: IgG and IgM concentration which reduced gently in a downward curve for > 35 days. Cytokines were also stimulated. IFN-Ɣ was increased remarkably on the same day as exposure, while TGF-β, IL-10 and IL-12 were also produced. The total population of B-cell and T-cell increased, decreasing the antigen. The T helper cell population rose, with an increase in both total and memory T-helper cells, and TH-1 cells. Additionally, B cell population per state (cells per mm^3^) demonstrated a decrease in active B cells upon injection and a gradual rise after 5 days before its most active state after approximately 8 days. Meanwhile, the T cell population per state (cells per mm^3^) observed a steady rise in active T cells after 5 days of injection. Overall, immune simulation results displayed active stimulation of role players of the immune system after vaccine dose, further validating our vaccine to be promising in protection against rotavirus.

The docking of the refined structure with an Integrin receptor subunit gave an insight into their interaction. The subunit of integrin was chosen based on the literature reporting its involvement during the entry of Rotavirus inside the cell. The weighted score of the center was −718.7 Kcal/mol and the lowest energy was −871 Kcal/mol.

To analyze the biophysical interactions between integrin and the vaccine construct, an MD simulation was conducted using GROMACS. The RMSD value of vaccine construct in the current study was slightly high (0.2-1.6 nm) which is comparable to a study conducted by Y.D Devi et al. [32]. This variation may be due to the nature of interactions between the two proteins, the ligand and receptor or trends can be followed further by increasing the time of simulation. In addition, RSMD values could vary depending on the type of molecules which are interacting in the complex (e.g., protein interacting with small ligands, peptides or protein–protein complexes), as well as the nature of molecular interactions. In general, RMSD values below 2 A (0.2 nm) are acceptable for docking with small ligands. On the other hand, protein–peptide docking could present higher RMSD values during the MD simulations due to the molecular accommodation of the peptide inside the protein pocket and in a given time (50 ns in this case) and dynamic changes in forming hydrogen and non-polar interactions between two molecules with higher molecular weights. In this case, the RMSD value should be interpreted in combination with other metrics such as binding affinity and biological relevance to fully assess the quality of a protein–peptide docking prediction [76,77,78]. On the other hand, integrin binding residue exhibited minimal fluctuations, indicating that the complex was stable, and these findings are comparable with previous studies [32,79].

## 5. Conclusions

Computer-aided vaccine design has emerged as a smart and promising approach for the development of vaccines. The present study utilized this approach by identifying B cell and T cell antigenic regions which were screened, linked by specific linkers and joined with an adjuvant to increase their immunogenicity. Through computational analysis the vaccine construct was found to be safe, non-allergenic and non-toxic when compared to live attenuated vaccines with known side effects: fever, vomiting, diarrhea and intussusception. Thus, theoretically, the proposed vaccine candidates are better and need further in vitro as well as in vivo testing to prove the computational results. Likewise, the results indicate the effectiveness of the multivalent vaccine in stimulating both the cell-mediated and humoral immune system. Although the findings of the current study are largely based on in silico predictions, the vaccine has the potential to stimulate the immune system. However, extensive wet lab/in vivo experiments using animal models and later clinical trials are required to validate the current results.

## Figures and Tables

**Table 1:** Detail of the selected epitopes and their different properties.

Protein	Epitope Type	Peptide	Vaxijen Score; ≥0.5	Allergenicity	IFNγ Induction	Toxicity	Anti- Inflammatory Response	Total Amino Acids	MW	pI
**VP4**	B cell	VTLSTQFTDFVSLNSLRFRF	1.1633	Non-allergen	+	Non-toxic	AIP	20	2378.71	9.57
		DSPVISAIIDFKTLKNLNDN	0.47	Non-allergen	+	Non-toxic	AIP	20	2217.5	4.43
		MSFDDISAAVLKTKIDMSTQ	0.8802	Non-allergen	+	Non-toxic	AIP	20	2201.53	4.43
	MHC-I	NMVYVRSLA	0.4292	Non-allergen	−	Non-toxic	AIP	9	1052.26	8.75
		YINNGLPPI	0.9097	Non-allergen	−	Non-toxic	AIP	9	1000.16	5.52
	MHC-II	VISAIIDFK	1.2685	Non-allergen	+	Non-toxic	AIP	9	1005.22	5.81
		ISAAVLKTK	1.2881	Non-allergen	+	Non-toxic	AIP	9	930.16	10
**VP7**	B cell	MDITLYYYQQTDEANKWISM	0.4852	Non-allergen	+	Non-toxic	AIP	20	2513.82	4.03
	MHC-I	YYYQQTDEA	0.5149	Non-allergen	+	Non-toxic	AIP	9	1180.19	3.67
	MHC-II	YYQQTDEAN	0.5459	Non-allergen	+	Non-toxic	AIP	9	1131.12	3.67

IFNγ = Interferon gamma, pI = Isoelectric point, MW = Molecular weight, MHC = Major histocompatibility complex, VP = Viral protein. AIP = Anti-inflammatory response.

**Table 2:** Population coverage of the vaccine construct.

MHC Class	Region	Coverage	Average Hit	PC90
**Combined**	South Asia	99%	2.33	1.81
	Worldwide	98.47%	2.82	1.72
	West Africa	87.35%	1.5	0.79
	East Africa	78.3%	1.27	0.46
	North Africa	74.03%	1.15	0.39
	Central Africa	59.05%	0.8	0.24

**Table 3:** Detail of the physiochemical properties of the vaccine construct.

	Toxicity	Total Amino Acids	MW (g/mol)	pI	Formula	Total Atoms	Approx ½ Life			Instability Index	Gravy Score	Stability Status
							Mammalian Cell (In Vitro)	Yeast (In Vivo)	*E. coli* (In Vivo)			
**With adjuvant**	Non-toxic	172	18878.3	8.6	C845H1311N217O263S5	2641	1 h	2 min	2 min	35.94	−0.291	Stable
**Without** **adjuvant**	Non-toxic	169	18550	8.61	C833H1291N211O258S5	2598	1 h	30 min	>10 h	36.91	−0.246	Stable

**Table 4:** Docking scores.

	Integrin			
	Cluster	Members	Representative	Weighted Score
**Balanced**	0	120	Center	−836.7
	0	120	Lowest Energy	−964.2
**Electrostatic favored**	0	45	Center	−919.9
	0	45	Lowest Energy	−998.7
**Hydrophobic Favored**	0	100	Center	−905.2
	0	100	Lowest Energy	−1049
**Van der Waals**	0	56	Center	−280.9
	0	56	Lowest Energy	−295.1

## Data Availability

All the data generated in this study is included in the current manuscript and in the Supplementary material and is not submitted to any database. Any specific data can be provided if needed.

## Supplementary Materials

The following supporting information can be downloaded at: https://www.mdpi.com/article/10.3390/vaccines11040726/s1, Figure S1: Adenoviral vector, pShuttle-CMV, construct carrying the gene of interest.; Figure S2: MD Simulation; Figure S3: Percent class combined coverage of vaccine construct in Africa.
