# Supplementary material for: Vaccinomics Approach for Multi-Epitope Vaccine Design against Group A Rotavirus Using VP4 and VP7 Proteins
Source: Vaccines (Basel). 2023 Mar 24;11(4):726. doi: 10.3390/vaccines11040726 (PMC10144065; doi:10.3390/vaccines11040726)
Supplement: Supplementary file 1 [file vaccines-11-00726-s001.zip › vaccines-2202569-supplementary.pdf]

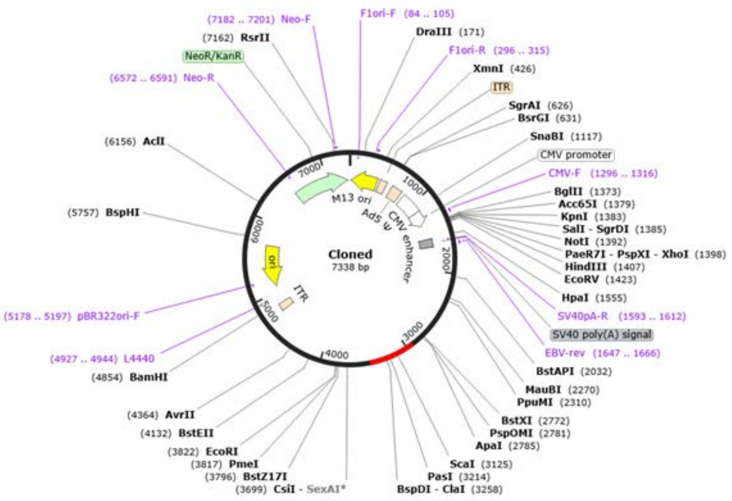

Figure S1. Adenoviral vector, pShuttle-CMV, construct carrying the gene of interest.

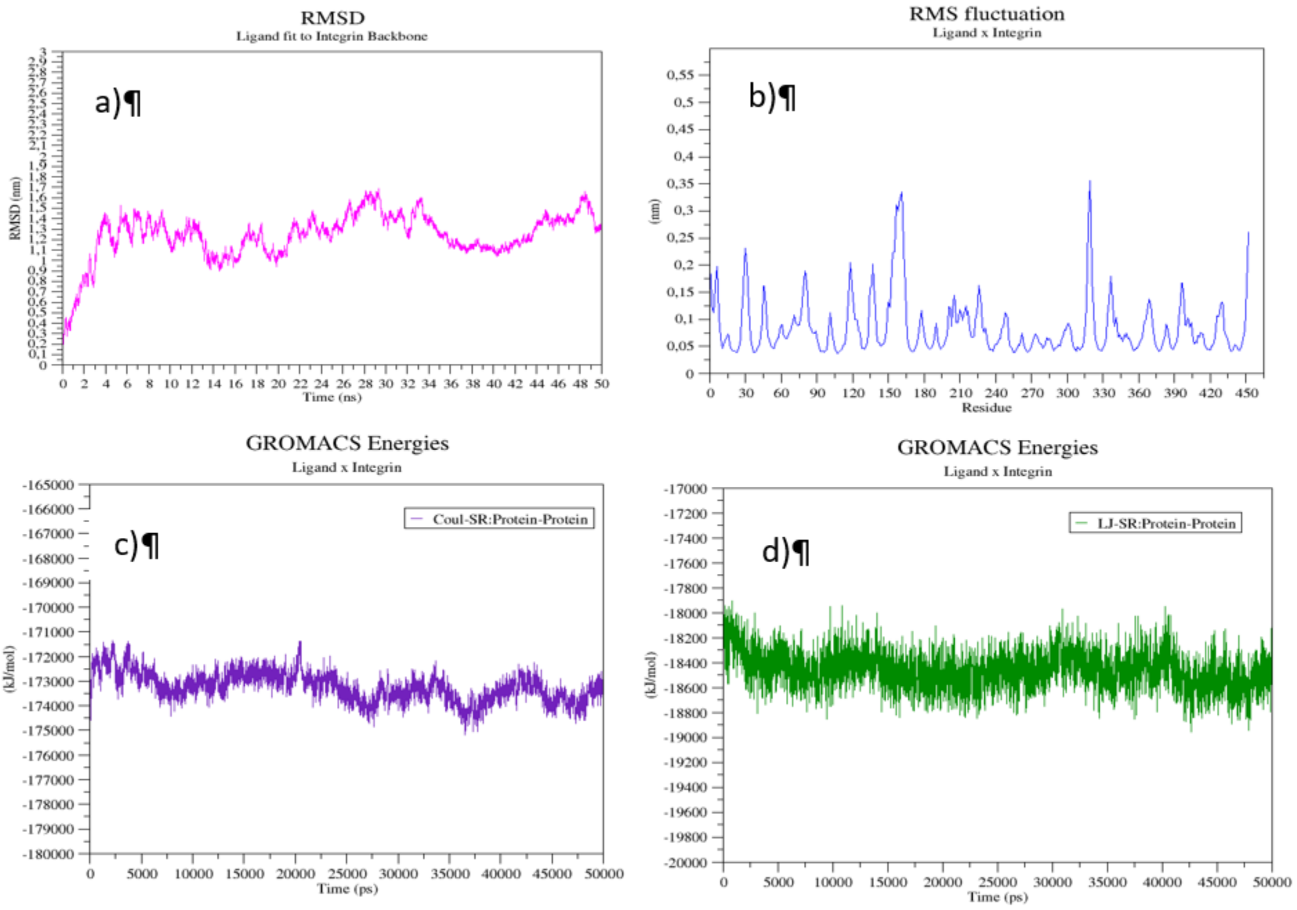

Figure S2. MD simulation. (**a**) Ligand backbone RMSD during the total MD simulation of 50ns interacting with the integrin structure. (**b**) Integrin amino acid fluctuation graph during the 50ns of interaction with its proposed ligand. (**c**) Short-range Coulomb energy graph during 50ns of MD simulation. (**d**) Short-range Lenard Jones energy graph during 50ns MD simulation.

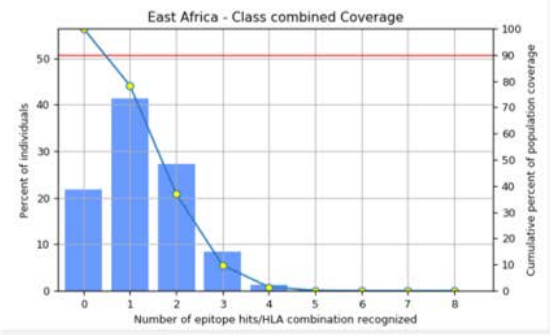

a)

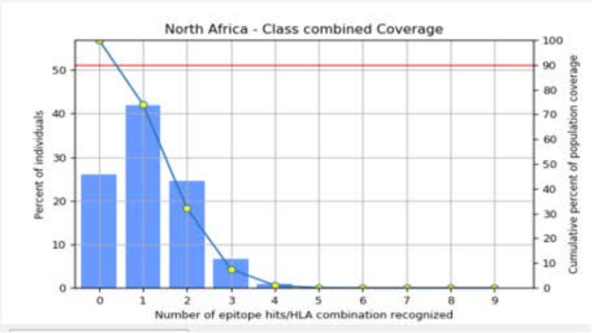

b)

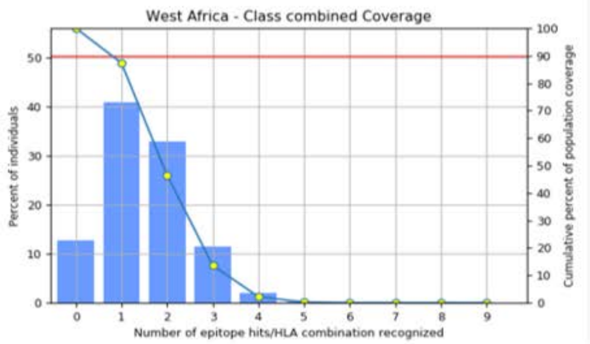

c)

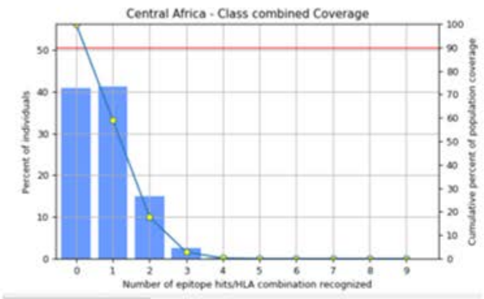

d)

Figure S3. Percent class combined coverage of the vaccine construct in Africa. (**a**) East Africa, (**b**) North Africa, (**c**) West Africa and (**d**) Central Africa.
